# A Pragmatic Bayesian Adaptive Trial Design Based on the Value of Information: The Value-Driven Adaptive Design

**DOI:** 10.1177/0272989X261423177

**Published:** 2026-03-13

**Authors:** Michael Dymock, Julie A. Marsh, Mark Jones, Anna Heath, Kevin Murray, Thomas L. Snelling

**Affiliations:** School of Population and Global Health, The University of Western Australia, Nedlands, WA, Australia; Wesfarmers Centre of Vaccines and Infectious Diseases, The Kids Research Institute Australia, Nedlands, WA, Australia; Wesfarmers Centre of Vaccines and Infectious Diseases, The Kids Research Institute Australia, Nedlands, WA, Australia; Centre for Child Health Research, The University of Western Australia, Crawley, WA, Australia; Sydney School of Public Health, University of Sydney, Camperdown, NSW, Australia; Child Health Evaluative Sciences, The Hospital for Sick Children, Toronto, Canada; Division of Biostatistics, Dalla Lana School of Public Health, University of Toronto, Toronto, Canada; Department of Statistical Science, University College London, London, UK; School of Population and Global Health, The University of Western Australia, Nedlands, WA, Australia; Sydney School of Public Health, University of Sydney, Camperdown, NSW, Australia

**Keywords:** value of information, bayesian, adaptive trial design, decision rules

## Abstract

**Background:**

Clinical trial designs are typically narrowly focused on error control in hypothesis testing, but this approach is inadequate in many contexts, particularly when a decision maker intends to, or must, consider multiple relevant clinical and health economic outcomes under uncertainty. Value-of-information (VoI) metrics can be used to estimate the monetary value of data collection to the decision maker. Adaptive trial designs use prespecified decision rules as data are collected and analyzed to modify the ongoing trial design. To date, VoI considerations have rarely been integrated into this approach, partly due to the computational burden.

**Methods:**

We propose a value-driven adaptive design that refocuses trial design on VoI as a metric to direct trial adaptations. Specifically, a VoI analysis is performed at each interim analysis to determine whether or not the trial should proceed to the next analysis (i.e., determine whether further data collection is sufficiently valuable). We provide methods to compute the expected net benefit of perfect information, expected net benefit of sampling (ENBS) for the next analysis, and the ENBS for subsequent sequential analyses. Our approach is flexible to any statistical model, decision model, and research cost function and does not require distributional assumptions about the net benefit.

**Results:**

We describe our method in detail and demonstrate its implementation via a case study comparing infant immunoprophylaxis and maternal vaccination to prevent respiratory syncytial virus–related medical attendances.

**Conclusions:**

Our value-driven adaptive design aligns pragmatic clinical trial design with the requirements of decision makers. Designs with VoI-based adaptations have the potential to improve the cost-effectiveness of clinical trials.

**Highlights:**

- Our value-driven adaptive design is a new method that uses the expected net benefit of sampling to define stopping rules at interim analyses (i.e., to determine if further data collection is sufficiently valuable).
- Our method orients trial designs to efficiently produce evidence to inform the decision maker.

Health care decision makers rely on high-quality evidence generated from clinical trials to guide their decision making. For example, in Australia, the Australian Technical Advisory Group on Immunisation makes clinical recommendations for the Australian Immunisation Handbook^1^ based on the aggregation of clinical evidence.^2^ Unfortunately, in some fields of research such as vaccines, despite aiming for high quality, evidence from clinical trials is often graded as being of low quality for translation to clinical practice recommendations, often due to a lack of precision in the estimated parameters for the reported outcomes.^3^

Determining a sample size that balances trial operating characteristics, feasibility, and ethical considerations is a challenge that faces all clinical trials. In general, trials are designed to ensure a reasonable chance of detecting and concluding, with sufficient certainty, a treatment benefit if one exists while mitigating against the risk of a false detection if a treatment benefit does not exist.^4^ Traditionally, a sample size that achieves acceptable trial operating characteristics with respect to a null hypothesis significance test relating to the primary outcome is computed using formulae or simulation. However, the error-driven approach to clinical trial design (i.e., the classical approach focused on type I and type II error control) is increasingly regarded as inadequate as it ignores the cost-effectiveness of the proposed research.^5,6^ Clinical trials could instead be designed and prioritized to optimize the net prospective monetary value of the evidence generated to clinical and policy decision makers (i.e., a value-driven approach).^6^

## Value of Information

Value-of-information (VoI) metrics such as the expected value of perfect information^7^ (EVPI), expected value of partial perfect information^8^ (EVPPI), and the expected value of sample information^9^ (EVSI) have been proposed as a way to assist researchers and funders during the trial design stage by providing information on the monetary value of conducting the proposed trial.^10,11^ Recent advances in approximation methods^12–20^ to compute these metrics have had their performance benchmarked,^21,22^ and practical guidance has been provided for their implementation.^23^ Specifically, these metrics allow researchers to quantify and compare the expected cost of conducting a particular study versus the expected monetary value gained from the study in order to inform a decision about whether the research should be funded and, if so, which design is the most cost-efficient.^24,25^

Typically, such analyses have been used to supplement, rather than replace, the sample size calculation.^5,26^ In principle, the sample size calculation seeks to determine the number of units required to identify a clinically meaningful effect under a given design, whereas the VoI analysis determines whether the proposed trial can be expected to be cost-effective (i.e., the monetary value of the evidence derived from the study outweighs the cost of research). However, the separation of these components might not be ideal. A proposed trial that is dismissed as “underpowered” according to convention might nonetheless be cost-effective from a VoI perspective.

### Adaptive Trial Designs

Adaptive trial designs are a popular alternative to traditional fixed trial designs.^27–29^ Instead of recruiting to a fixed prespecified sample size, adaptive designs incorporate interim analyses that apply prespecified decision rules based on model parameter estimates. Interim analysis decision rules guide the ongoing design of the trial to allocate resources efficiently or to stop early if a trial conclusion can be declared.^30,31^

VoI analyses are rarely incorporated into adaptive designs in practice,^32^ partly due to concerns^33^ about the complexity of the analyses required, although there has been recent methodological progress. Vervaart et al.^34^ developed methods to assess the value of collecting additional survival data by extending the follow-up period of currently enrolled trial participants to inform treatment adoption decisions within health economic evaluations. These methods are described with practical guidance in a tutorial^35^ and may be implemented at interim analyses as value-based stopping rules. Flight et al.^36^ adapted the EVSI calculation for group sequential designs with clinical effectiveness stopping rules and suggested that VoI methods could be used at interim analyses to inform the ongoing trial design but that computing the EVSI one analysis at a time “does not take account of all possible future interim analyses.” Griffin et al.^37^ described how to compute the VoI for designs that resolve uncertainty in parameter sets sequentially using the EVPPI. However, computational complexity was identified as a potential barrier to the sequential EVSI extension of these methods.^37^

Decision-theoretic models using dynamic programming methods have also been proposed for adaptive trial designs,^38–41^ for example, a Bayesian group sequential design based on a quadratic loss function,^41^ and these methods have been subsequently extended.^42–45^ More recently, building on the work of Pertile et al.,^44^ a decision theoretic model has been extended to account for delay in outcome ascertainment,^46^ for multiple trial interventions,^47^ and to select optimal recruitment rates^48^ with 2 retrospective case-study applications^49,50^ and a tutorial.^51^ These models and their contemporaries estimate the distribution of the expected incremental net monetary benefit (INMB; the incremental monetary value of one decision over another) using trial-based economic evaluations (where the INMB is estimated using only trial data). Under certain assumptions, for example, a Gaussian prior distribution and likelihood, optimal stopping boundaries may be derived analytically and optimal allocation policies prescribed.

### Aim

Trial-based economic evaluations may be inappropriate if all available treatment options considered by the decision maker are not included, the trial time horizon is truncated, or there is a failure to incorporate all relevant evidence.^52,53^ Sculpher et al.^52^ suggested that trials should not be considered as the “vehicle for economic evaluation” but should instead generate evidence to be synthesized into the full, externally specified decision model (e.g., by reducing parameter uncertainty). To align with Sculpher et al.,^52^ we propose a new method that incorporates VoI methods into Bayesian adaptive trial designs based on a complete decision model. We aim to orient trial designs to efficiently produce evidence to inform the decision maker by conducting VoI analyses as part of the trial’s prespecified interim analyses. Specifically, a VoI analysis based on the expected net benefit of sampling (ENBS) will be performed at each interim analysis to determine if the trial should proceed through to the next analysis (i.e., determine if further data collection is sufficiently valuable).

Our method, termed the *value-driven adaptive design*, may be considered in the class of value-adaptive designs^51^ and intends to offer an alternative pragmatic approach to the aforementioned decision-theoretic models and contemporaries. Our approach accommodates any Bayesian statistical model (e.g., a hierarchical model) and allows for the decision model and statistical model to be specified independently. Building on the work of Griffin et al.,^37^ and providing an alternative to Pertile et al.,^44^ our value-driven adaptive design is the first adaptive design using a VoI-based decision rule at interim analyses to determine the value of continuing recruitment to repeatedly reduce parameter uncertainty without restriction or distributional assumptions on the statistical model or net benefit. Our method is general purpose and not restricted to any specific health economic model structures (as required by Pertile et al.^44^) or approximation algorithms (i.e., our method can use regression based approximation algorithms^18^ that maintain computational complexity, irrespective of the complexity of the health economic model). The article proceeds as follows: the second section describes our methodology in detail, the third section presents a case study as a motivating example, and the fourth section concludes with a discussion.

## Methodology

Suppose that a decision maker was choosing between an exhaustive choice set of D decision options based on a decision model represented by the utility function $\mathrm{NB}\left(d,\Theta ,t_{1},t_{2}\right)$ , expressed as the population-level net benefit and specified by the decision maker independently of any research design. The model depends on decision option d , where d∈{1,2,…,D} , parameters Θ and times $t_{1}$ and $t_{2}$ with $0\leq t_{1}<t_{2}$ . Times $t_{1}$ and $t_{2}$ specify the time period for which the population of interest accrues (possibly discounted) value from the decision options. Usually, $t_{1}=0$ represents the current time and $t_{2}=t_{H}$ where $t_{H}$ is the decision horizon (e.g., the length of time before the decision would be reassessed). We specify the decision model at the population level to allow for scenarios in which the simple scaling of an individual-level model by the population size may be inappropriate (e.g., a vaccine model incorporating indirect benefits from herd immunity). The net benefit function is specified with respect to (health payer or societal) monetary benefits and costs. We introduce benefit function $B\left(d,\Theta ,t_{1},t_{2}\right)$ and cost function $C\left(d,\Theta ,t_{1},t_{2}\right)$ , to express the net benefit function as follows:

(1) $$\begin{array}{c} \mathrm{NB}\left(d,\Theta ,t_{1},t_{2}\right)=\lambda \times B\left(d,\Theta ,t_{1},t_{2}\right)-C\left(d,\Theta ,t_{1},t_{2}\right) \end{array}$$

Here, λ is the fixed and usually, but not necessarily, assumed willingness-to-pay parameter that expresses the benefits on the same monetary scale as the costs. The benefit function may contain parameters representing an intervention’s effectiveness, often with respect to a utility measure such as quality-adjusted life-years (QALYs), whereas the cost function may contain parameters representing the intervention costs (costs of implementation, health consequences, etc.). A clinical trial will typically collect information on the parameters of the benefit function (i.e., investigate the clinical effectiveness of an intervention) and sometimes on some of the parameters of the cost function.

If the parameters Θ were known with certainty, then the optimal decision would be the one that maximized the net benefit function (1). However, there is usually uncertainty in the parameters (e.g., represented via prior distributions), which propagates through to uncertainty in the net benefit function. If required to make a decision now, a rational risk-neutral decision maker would choose the decision option that maximized the expectation of the net benefit function over the parameter uncertainty (often referred to as the *expected value given current information*).

### A Clinical Trial

Suppose we were to conduct a clinical trial with sequential analyses in which we accrue information on unit outcomes and progressively resolve epistemic uncertainty in the value of the net benefit function (1) (which could be exploited by the decision maker to make an improved decision). Our goal is to progress through the sequential analyses until it is no longer valuable. We denote all relevant outcome data collected for participant i∈{1,2,…,N} , where N is the maximum sample size, with $X_{i}$ (note that we use lowercase $x_{i}$ to denote the data once observed). Between analysis j−1 and j , with analyses denoted j∈{1,2,…,J} occurring at ascending times $t_{j}$ where $0<t_{j}<t_{H}$ , data are collected for $n_{j}$ participants, where $\sum_{j=1}^{J}n_{j}=N$ (with no requirement to prespecify N or J ). At analysis j , we denote the number of participants recruited thus far with $N_{j}=\sum_{j^{*}=1}^{j}n_{j^{*}}$ (where $N_{J}=N$ ), the currently observed data with $x^{j}=\{x_{1},x_{2},\ldots ,x_{N_{j}}\}$ and yet to be collected data on the next $n_{j+1}$ participants with $X^{\;j+1}=\{X_{N_{j}+1},X_{N_{j}+2},\ldots ,X_{N_{j+1}}\}$ , which may be generated via the posterior predictive distribution.

The function η(j) represents the cost of data collection to proceed from analysis j−1 through to analysis j and should be defined to capture all of the relevant costs (e.g., recruitment, outcome ascertainment and statistical resources). Although in principle there is no restriction on the form of the function, we define an example cost function below where δ is the fixed start-up cost of the trial and γ is the constant per participant cost:

(2) $$\begin{array}{c} \eta \left(j\right)=\left\{ \begin{array}{cc} \delta +\gamma \times n_{j} & \mathrm{if}\;j=1\\ \gamma \times n_{j} & \mathrm{if}\;j\;>1 \end{array}\right. \end{array}$$

### Expected Net Benefit of Sampling

We denote the assumed implementation of decision options during the trial with $p=\{p_{1},p_{2},\ldots ,p_{D}\}$ as the unit D -simplex, where $p_{d}$ represents the current proportion of the general population of interest receiving decision option d . The total value added through data collection can be conceptualized as the sum of the value accrued in the general population during the data collection (the current implementation of the interventions) and the value accrued in the population following data collection (from the implementation of a potentially improved decision). While we acknowledge that implementation considerations are not usually included in VoI definitions, we include them here to account for the potential costs associated with imperfect implementation while the research is conducted. As clinical trials are usually long, this may substantially influence the potential value of research. One should proceed with data collection only if the expected value accrued exceeds the expected value given current information and the cost of data collection. Thus, we formally define the ENBS between analyses j and j+1 computed at time $t_{j}$ , denoted $\mathrm{ENB}\;S^{j}$ (which is implicitly conditional on the observed data ${x\;}^{j}$ ):

(3) $$\begin{array}{c} \begin{array}{c} \mathrm{ENB}\;S^{j}=E_{\Theta |{x\;}^{j}}\left[\sum_{d=1}^{D}p_{d}\times \mathrm{NB}\left(d,\Theta ,t_{j},t_{j+1}\right)\right]+\\ \;E_{X^{\;j+1}|{x\;}^{j}}\left[\underset{d}{\max\;}E_{\Theta |{x\;}^{j},X^{\;j+1}}[\mathrm{NB}\left(d,\Theta ,t_{j+1},t_{H}\right)]\right]-\\ \;\left(\underset{d}{\max\;}E_{\Theta |{x\;}^{j}}\left[\mathrm{NB}\left(d,\Theta ,t_{j},t_{H}\right)\right]+\eta \left(j+1\right)\right) \end{array} \end{array}$$

**Figure 1:**
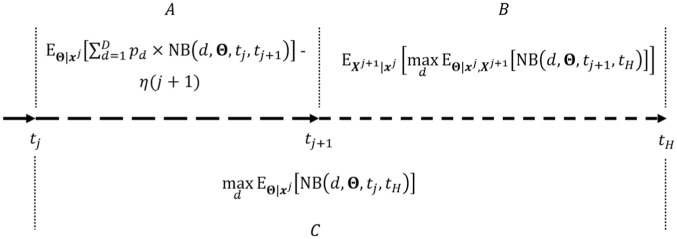
Visual representation of the expected net benefit of sample information ( $\mathrm{ENB}S^{j}$ ). A decision is made at time $t_{j}$ to either continue recruitment to time $t_{j+1}$ (panels A and B) or to stop the trial (panel C). (A) Between time $t_{j}$ and $t_{j+1}$ (while the research is conducted), value is accrued to the general population according to the current implementation of decision options ( p ) and the cost of conducting the research ( η(j+1) ) is spent. (B) Between time $t_{j+1}$ and $t_{H}$ (after the research is conducted), value is accrued to the population with the implementation of the best decision option given updated information. (C) Between time $t_{j}$ and $t_{H}$ (instead of conducting research), value is accrued to the population with the implementation of the best decision option given current information.

The ENBS equation (3) is visualized in Figure 1. If $\mathrm{ENB}\;S^{j}\;>\;\epsilon$ , where ϵ is the stopping rule threshold and usually equal to zero, and we are not at the final analysis, then we proceed through to analysis j+1 and compute $\mathrm{ENB}\;S^{\;j+1}$ because further research is sufficiently valuable. We note here that once analysis j+1 has been conducted, the values of $\mathrm{ENB}\;S^{j^{\;'}}\;\forall j^{\;'}<j+1$ become obsolete, and only $\mathrm{ENB}\;S^{\;j+1}$ is of interest. If $\mathrm{ENB}\;S^{j}\leq \epsilon$ or we are at the final analysis, then we stop recruitment. We assume that we wait for the current participants to complete follow-up before choosing a decision option. However, a decision option may instead be chosen before observing the follow-up data if there is sufficient justification (e.g., by ignoring incomplete follow-up data or predicting it). However, this decision is itself nontrivial, context dependent, and influenced by the decision maker’s risk aversion to the follow-up data conflicting with the already chosen decision option.

### Expected Net Benefit of Perfect Information

Analogous to the EVPI, we define the expected net benefit of perfect information (ENBP) as the expected value of proceeding through to the next analysis under the assumption that doing so will resolve all parameter uncertainty. The ENBP computed at analysis j , denoted $\mathrm{ENB}\;P^{j}$ and also implicitly conditional on the observed data ${x\;}^{j}$ , is

(4) $$\begin{array}{c} \begin{array}{c} \mathrm{ENB}\;P^{j}=E_{\Theta |{x\;}^{j}}\left[\sum_{d=1}^{D}p_{d}\times \mathrm{NB}\left(d,\Theta ,t_{j},t_{j+1}\right)\right]+\\ \;E_{\Theta |{x\;}^{j}}\left[\underset{d}{\max\;}\mathrm{NB}\left(d,\Theta ,t_{j+1},t_{H}\right)\right]-\\ \;\left(\underset{d}{\max\;}E_{\Theta |{x\;}^{j}}\left[\mathrm{NB}\left(d,\Theta ,t_{j},t_{H}\right)\right]+\eta \left(j+1\right)\right) \end{array} \end{array}$$

The expression for $\mathrm{ENB}\;P^{j}$ (4) is computationally simpler than $\mathrm{ENB}\;S^{j}$ (3) because the inner expectation within the second term is no longer required (i.e., we do not need to estimate the uncertainty reduction due to observing potential future data because there is no longer any uncertainty). The $\mathrm{ENB}\;P^{j}$ is an upper bound for $\mathrm{ENB}\;S^{j}$ , and so if $\mathrm{ENB}\;P^{j}\leq \epsilon$ , then we may stop the trial without needing to compute $\mathrm{ENB}\;S^{j}$ (i.e., if perfect information is not sufficiently valuable, then sample information will not be).

### Expected Net Benefit of Continuing the Trial

The above expression for $\mathrm{ENB}\;S^{j}$ (3) computes the expected value of proceeding through to the next analysis and then implementing the “best” decision option (i.e., stopping the trial and making a decision). If one were instead interested in computing the expected value of proceeding through to the next analysis with the possibility of continuing the trial to one more analysis ( j+2 ), then a different quantity, which we denote $\mathrm{ENB}\;S^{j,j+1}$ , is required. Denoting $\mathrm{ENB}\;S^{\;j+1}|X^{\;j+1},{x\;}^{j}$ as the random variable for the ENBS estimated at analysis j+1 given the currently observed data ${x\;}^{j}$ and potential unobserved data $X^{\;j+1}$ , the expression for $\mathrm{ENB}\;S^{j,j+1}$ is

(5) $$\begin{array}{c} \mathrm{ENB}\;S^{j,j+1}=\mathrm{ENB}\;S^{j}+E_{X^{\;j+1}|{x\;}^{j}}\left[\max\left\{\left(\mathrm{ENB}\;S^{\;j+1}|X^{\;j+1},{x\;}^{j}\right),0\right\}\right] \end{array}$$

A proof for equation (5) is provided in the Supplementary Materials. In (5), the inner part of the second term represents the decision on whether or not to proceed to analysis j+2 from analysis j+1 via the expression $\mathrm{ENB}\;S^{\;j+1}$ conditional on a set of potential future data $X^{\;j+1}$ and the currently observed data ${x\;}^{j}$ . For each potential set of future data $X^{\;j+1}$ , we proceed to analysis j+2 only if $\mathrm{ENB}\;S^{\;j+1}\;>\;0$ , and so the second term of the equation is necessarily nonnegative. Equation (5) can be extended recursively to estimate the expected value of proceeding to a second further analysis ( j+3 ) and so on through to the end of the trial ( J ). Estimation of $\mathrm{ENB}\;S^{j,j^{\;'}}$ (5), where $j^{\;'}\;>\;j$ , is computationally demanding, if not infeasible, for all but the simplest of trial designs, especially when $j^{\;'}>>j$ . The computational complexity has order of approximation $O\left(s^{\;j^{\;'}-j}\right)$ , where s is the number of draws from the posterior predicted distribution (or similar) used to simulate potential future data and $j^{\;'}-j$ is the number of potential future analyses.

Furthermore, $\mathrm{ENB}\;S^{j}\leq \mathrm{ENB}\;S^{j,j^{\;'}}<\mathrm{ENB}\;P^{j}$ , where $\mathrm{ENB}\;S^{j}$ and $\mathrm{ENB}\;P^{j}$ are relatively computationally cheaper to compute and therefore provide convenient lower and upper bounds, respectively. $\mathrm{ENB}\;S^{j}$ also converges to $\mathrm{ENB}\;S^{j,j^{\;'}}$ as j increases as there are fewer remaining potential analyses and the expected potential uncertainty reduction decreases (i.e., more uncertainty is resolved in the early analyses). Moreover, $\mathrm{ENB}\;S^{j,j^{\;'}}$ is of practical interest only when $\mathrm{ENB}\;S^{j}<\epsilon <\mathrm{ENB}\;P^{j}$ (i.e., it does not appear valuable to proceed to collect sample information up to the next analysis but it does appear valuable to collect perfect information and potentially valuable to collect more sample information via $\mathrm{ENB}\;S^{j,j^{\;'}}$ ). In these instances, $\mathrm{ENB}\;S^{j,j+1}$ and $\mathrm{ENB}\;S^{j,j+2}$ offer less computationally intensive approximations to $\mathrm{ENB}\;S^{j,j^{\;'}}$ where $j^{\;'}\;>\;>j$ and may be estimated if desired (e.g., by considering the value of proceeding through to analysis j+1 and possibly to j+2 but no further).

### Choosing Interim Sample Sizes

Our value-driven adaptive design can be used irrespective of the method for selecting interim sample sizes. Many trials choose interim sample sizes based on practical constraints such as anticipated recruitment, availability of statistical resources and the length, cost, and feasibility of outcome ascertainment (e.g., laboratory-based analyses may require batched samples). In these cases, we advise choosing interim sample sizes pragmatically.

**Figure 2:**
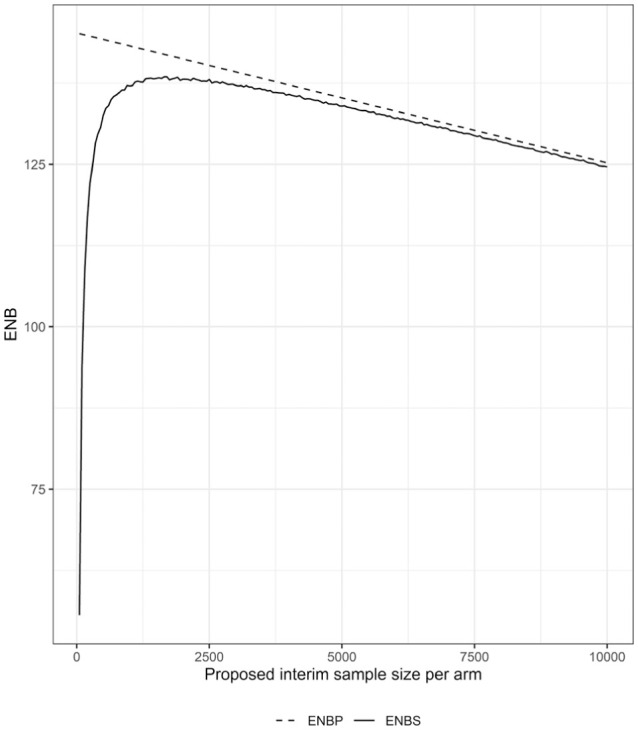
Expected net benefit (ENB) of perfect information (ENBP) and sampling information (ENBS) by proposed interim trial sample size per arm. Proposed interim sample sizes range between 50 and 10,000 participants per arm.

Alternatively, interim sample sizes may be chosen strategically at each interim analysis by choosing $n_{j}$ to maximize $\mathrm{ENB}\;S^{j}$ . As an example, consider $\mathrm{ENB}\;S^{j}$ estimated for a range of potential interim sample sizes in Figure 2. Here, although $\mathrm{ENB}\;S^{j}\;>\;\epsilon =0$ for all of the proposed interim sample sizes, it is maximized for interim samples sizes between 1,000 and 2,500. One may conservatively choose smaller sample sizes to allow for more opportunities to use the data to guide the ongoing trial design (e.g., by reassessing the modeling assumptions) or choose larger sample sizes that still produce trials of net benefit if the statistical and laboratory resources required for multiple analyses are constrained; the extreme of this would be to choose a fixed trial design.

### Summary

Having presented the key measures to define our value-driven adaptive design, we provide a summary of the steps in Box 1. We also provide generic code that implements our method by calculating $\mathrm{ENB}\;S^{j}$ (3), $\mathrm{ENB}\;P^{j}$ (4), and $\mathrm{ENB}\;S^{j,j^{\;'}}$ (5) and our case study in an R package^54^ available at https://github.com/michaeldymock25/ValueAdapt.

**Box 1:** 

1. Specify the decision model, associated net benefit function $\mathrm{NB}\left(d,\Theta ,t_{1},t_{2}\right)$ , threshold ϵ , and statistical model. 2. Set j=0 . 3. Compute $\mathrm{ENB}\;P^{j}$ for the proposed trial design recruiting $n_{j+1}$ participants. (a) If $\mathrm{ENB}\;P^{j}<\epsilon$ , stop and choose the decision option that maximizes the expected value of the net benefit function given current information. (b) Otherwise, proceed to step 4. 4. Compute $\mathrm{ENB}\;S^{j}$ for the proposed trial design recruiting $n_{j+1}$ participants. (a) If $\mathrm{ENB}S^{j}<\epsilon$ , stop and choose the decision option that maximizes the expected value of the net benefit function given current information. (b) Otherwise, proceed to step 5. 5. Recruit $n_{j+1}$ participants, increment j←j+1 , and repeat steps 3 to 5 for each interim analysis or until maximum recruitment (if specified).

## Case Study

To demonstrate our value-driven adaptive design, we consider an example trial evaluating infant immunoprophylaxis (II) compared with maternal vaccination (MV) to prevent respiratory syncytial virus (RSV). RSV infection accounts for approximately 3.6 million hospitalizations and more than 100,000 deaths each year, globally.^55^ In Australia, the 2 potential alternative strategies to reduce the burden of severe RSV disease are II ( d=1 ) and MV ( d=2 ), and it is currently unknown which strategy would be more cost-effective if implemented. There are uncertainties regarding their potential uptake and coverage, protection against moderate and severe disease, and duration of protection. To simplify this case study, we ignore the effect of disease transmission and assume that II is the current implemented standard practice with 100% uptake (i.e., p={1,0} ).

### Net Benefit Function

Of interest to the policy maker is the tradeoff between the difference in effectiveness of II compared with MV with respect to preventing RSV-related medical attendances (MA-RSV) in the first 12 months of life and the respective costs of implementing each strategy. We denote the probability that infants belonging to mother–infant dyads who received II or MV experience an MA-RSV within the first 12 months with $p_{\mathrm{II}}$ or $p_{\mathrm{MV}}$ , respectively, where $\Theta =\{p_{\mathrm{II}},p_{\mathrm{MV}}\}$ .

We assume that in Australia, there are, on average, 300,000 births per year, the willingness to pay to prevent each MA-RSV is λ, and that there are no other RSV-related health care costs (i.e., we ignore costs of parental work absenteeism and potential long-term respiratory consequences of RSV infection). The individual total costs, in Australian dollars, for implementing II and MV are assumed to be $590 and $330, respectively. Expressed in terms of the incremental benefit of switching from II to MV per 1 million Australian dollars and considering a $t_{H}=15$ -year decision horizon with an annual discount rate of 5%, the population-level INMB is

(6) $$\begin{array}{c} \mathrm{INM}B\left(\Theta ,t_{1},t_{2}\right)=\frac{1}{1,000,000}\times \sum_{t=t_{1}}^{t_{2}-1}1.05^{-t}\times 300,000\times \left(\lambda \left(p_{\mathrm{II}}-p_{\mathrm{MV}}\right)+260\right) \end{array}$$

Note that we use a discrete approximation for the discounting function here only for the purpose of exposition and accessibility. In practice, at least for this example, a continuous discounting function may be more accurate and computationally advantageous. We see here that MV will be more cost-effective than II if $p_{\mathrm{MV}}-p_{\mathrm{II}}<\frac{260}{\lambda }$ (assuming that there are no costs to change the strategy from II to MV). We assume the willingness-to-pay parameter to be λ=$5,200 so that MV will be preferred if $p_{\mathrm{MV}}-p_{\mathrm{II}}<5\%$ and II will be preferred otherwise.

### The Trial

Suppose that a potential trial comparing the strategies head to head aims to recruit up to a maximum of 2,000 mother–infant dyads, denoted $i_{k}$ , randomized to strategies k∈{II,MV} and is estimated have a fixed startup cost ( δ ) of $1 million and a fixed per-dyad cost ( γ ) of $2,000 paid upon randomization. Dyad $i_{k}$ has outcome $x_{i_{k}}$ , where $x_{i_{k}}=1$ if the infant from dyad $i_{k}$ experiences an MA-RSV within the first 12 months and $x_{i_{k}}=0$ otherwise. The trial is group sequential with cohorts of 500 dyads (i.e., 250 dyads per strategy) recruited every 12 months. Analyses are conducted once each cohort has been recruited and infants have turned 12 months old (i.e., analyses j∈{1,2,3,4} are conducted after 2, 3, 4, and 5 years, respectively). The following binomial model is implemented at analysis j :

(7) $$\begin{array}{c} \sum_{i_{k}=1}^{N_{\mathrm{jk}}}X_{i_{k}}\sim \mathrm{Binomial}\left(N_{\mathrm{jk}},p_{k}\right)\;\forall k\in \{\mathrm{II},\mathrm{MV}\} \end{array}$$

We use regularizing Beta(4,20) priors for both unknown parameters $p_{\mathrm{II}}$ and $p_{\mathrm{MV}}$ , consistent with a probability distribution centered on 0.17 and with 95% of the distribution between 0.05 and 0.34. For illustrative purposes, we assume that the only relevant unknown parameters are $p_{\mathrm{II}}$ and $p_{\mathrm{MV}}$ and that the trial will provide information to reduce their uncertainty. In most applications, there will be a number of unknown parameters, and any given trial is likely to only provide information to reduce the uncertainty for a subset of those parameters. We make this simplifying assumption because the purpose of the case study is only to demonstrate our methodology, for which a simple example is best suited. In principle, our methodology is not restricted by the complexity of the decision model or statistical model, the number of decision options, or the proposed trial design (although, increased complexity in these factors will likely increase the computational demand). We set the threshold to proceed between analyses to ϵ=0 .

### Scenarios

To demonstrate the implementation of our method on the RSV case study, we consider the following 2 scenarios:

1. The incremental effectiveness of II compared with MV is large (i.e., II is preferred to MV). We set $p_{\mathrm{II}}=0.10$ and $p_{\mathrm{MV}}=0.18$ .
2. The incremental effectiveness of II compared with MV is small (i.e., MV is preferred to II). We set $p_{\mathrm{II}}=0.10$ and $p_{\mathrm{MV}}=0.12$ .

These parameters are fixed and known only for the purpose of data simulation. Prior to conducting a trial with an adaptive design, this method can be used within a simulation study to explore the trial’s potential course given current parameter uncertainty and does not require fixed truth scenarios such as those presented here.

### Implementation

Prior to starting the trial, we conduct a cost-effectiveness analysis to determine whether conducting the trial up to the first analysis is cost-effective (i.e., compute $\mathrm{ENB}\;S^{0}$ and $\mathrm{ENB}\;P^{0}$ via equations [3] and [4], respectively). The uncertainty in the parameters prior to the trial starting is represented by the prior distributions. We compute $\mathrm{ENB}\;P^{0}$ and use the regression method of Strong et al.^18^ to estimate the second term of $\mathrm{ENB}\;S^{0}$ . In principle, any approximation method may be used and should be chosen dependent on the context. Here, $\mathrm{ENB}\;P^{0}\approx \$145\;\mathrm{million}\;>\;\epsilon =0$ and $\mathrm{ENB}\;S^{0}\approx \$120\;\mathrm{million}\;>\;0$ , and so the trial proceeds.

**Figure 3:**
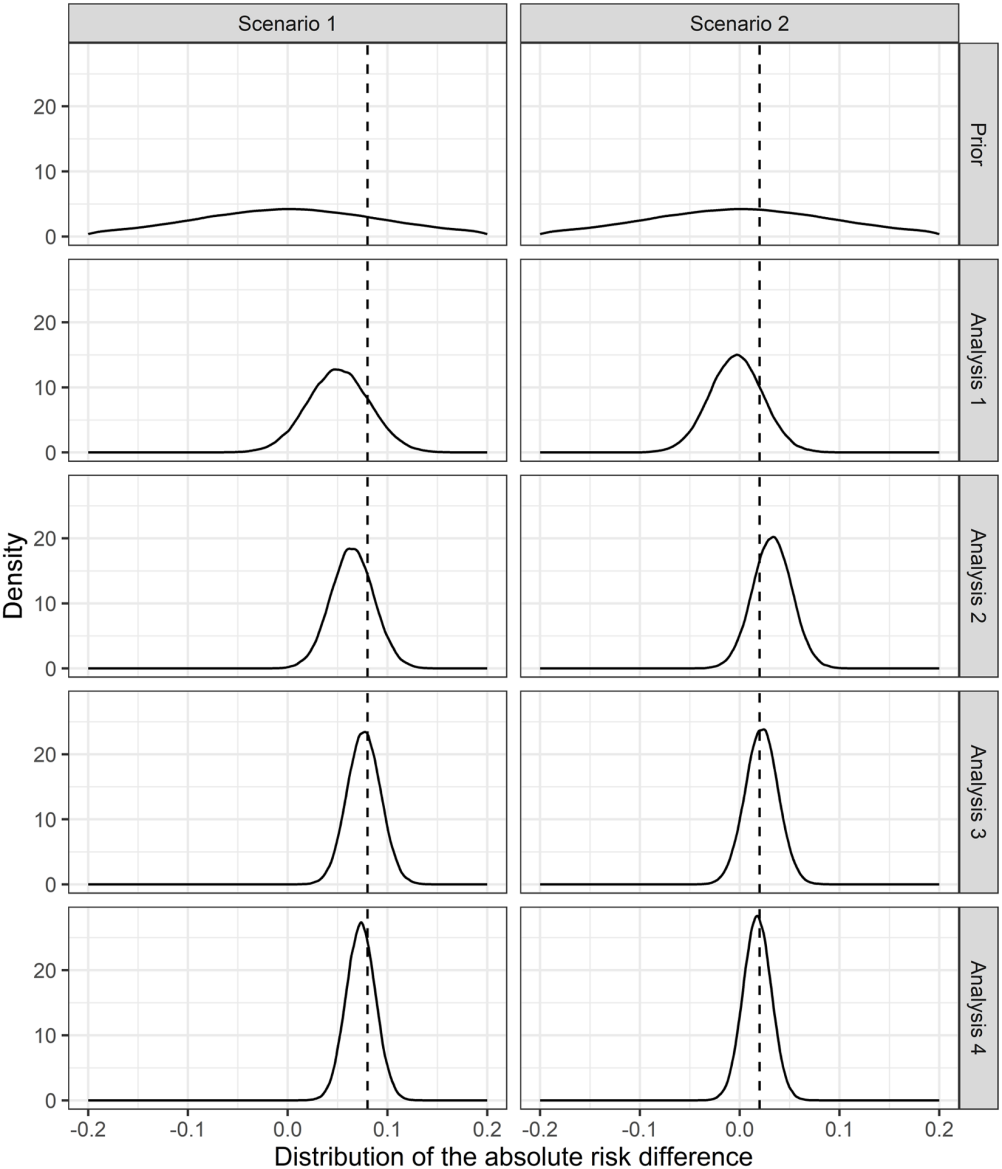
Distributions of the absolute risk difference of a medically attended respiratory syncytial virus event within 12 months between maternal vaccination and infant immunoprophylaxis ( $p_{\mathrm{MV}}-p_{\mathrm{II}}$ ) prior to the trial (prior distributions) and at each analysis (posterior distributions) for one simulated trial under each scenario of the respiratory syncytial virus case study. The dashed lines represent the true risk differences used for simulation.

**Figure 4:**
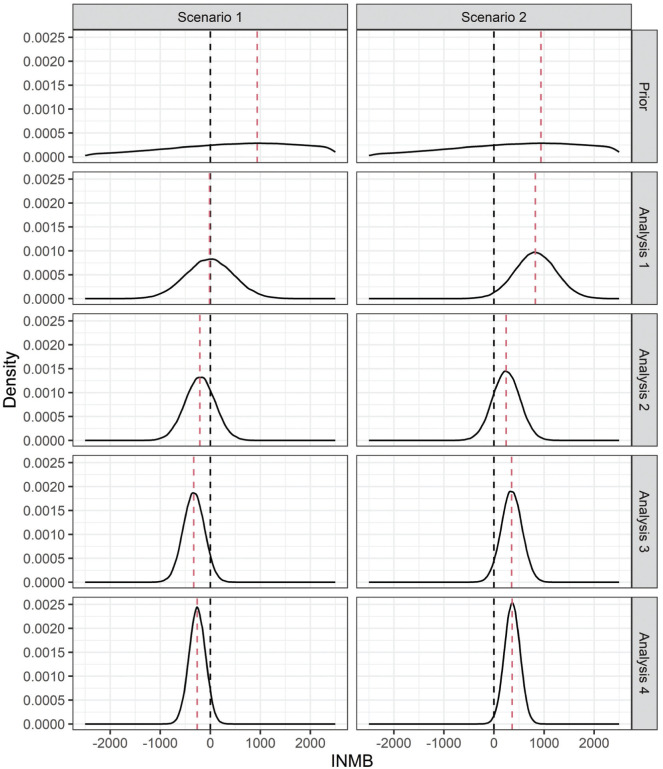
Incremental net monetary benefit (INMB) distributions prior to the trial (prior distributions) and at each analysis (posterior distributions) for one simulated trial under each scenario of the respiratory syncytial virus case study. The black dashed line at zero represents the decision point between the infant immunoprophylaxis and maternal vaccination strategies. The red dashed lines represent the expected INMB.

For each scenario, we generate data for each analysis (using equation [7]) and estimate the corresponding posterior distributions. As data accrue, posterior parameter uncertainty reduces, and consequently, uncertainty in the INMB (6) reduces too. Figures 3 and 4 show the uncertainty reduction in the parameters (as the absolute risk difference) and INMB, respectively, for each scenario and analysis for one set of simulated trials.

We estimate the value of potentially proceeding through to analysis j+2 (instead of only to j+1 , via $\mathrm{ENB}\;S^{j,j+1}$ ) and compute $\mathrm{ENB}\;P^{j}$ , $\mathrm{ENB}\;S^{j},$ and $\mathrm{ENB}\;S^{j,j+1}$ at each analysis in each scenario for this set of simulated trials (Table 1). Here we see that the trial in scenario 1 would have recruited until completion, whereas the trial in scenario 2 would have stopped recruitment at the first analysis. In each instance, $\mathrm{ENB}\;S^{j,j+1}$ is larger than but still close to $\mathrm{ENB}\;S^{j}$ (except for analysis 1 in scenario 2, where $\mathrm{ENB}\;S^{j,j+1}<\mathrm{ENB}\;S^{j}$ due to approximation error) and the analysis decisions were consistent. $\mathrm{ENB}\;S^{j,j+1}$ also required a considerably longer run time compared with $\mathrm{ENB}\;S^{j}$ (approximately 3 days compared with 5 seconds).

**Table 1:** Expected Net Benefit of Perfect Information ( $\mathrm{ENB}\;P^{j}$ ), Sampling Information ( $\mathrm{ENB}\;S^{j}$ ), and Sampling Information with the Possibility of Continuing to One Additional Further Analysis ( $\mathrm{ENB}\;S^{j,j+1}$ ) with Run Times for One Simulated Trial in 1 Million Australian Dollar Units for the Respiratory Syncytial Virus Case Study^a^

		Scenario 1		Scenario 2	
Analysis	Measure	Value	Run Time (min)	Value	Run Time (min)
Trial start	$\mathrm{ENB}\;P^{j}$	143.23	0.009	143.23	0.009
	$\mathrm{ENB}\;S^{j}$	120.75	0.061	120.75	0.061
	$\mathrm{ENB}\;S^{j,j+1}$	142.44	4,273	142.44	4,273
Analysis 1	$\mathrm{ENB}\;P^{j}$	148.50	0.005	−73.72	0.008
	$\mathrm{ENB}\;S^{j}$	100.60	0.093	−76.65	0.131
	$\mathrm{ENB}\;S^{j,j+1}$	111.93	4,289	−77.52	4,237
Analysis 2	$\mathrm{ENB}\;P^{j}$	33.67	0.004	−2.03	0.006
	$\mathrm{ENB}\;S^{j}$	6.09	0.083	−21.31	0.110
	$\mathrm{ENB}\;S^{j,j+1}$	12.39	4,271	−17.33	4,691
Analysis 3	$\mathrm{ENB}\;P^{j}$	4.18	0.004	−32.88	0.010
	$\mathrm{ENB}\;S^{j}$	0.02	0.063	−36.00	0.102
	$\mathrm{ENB}\;S^{j,j+1}$	—	—	—	—

a Between the start of the trial and analysis 1, there are 1,000 participants recruited. Between analyses 1 to 2 and 2 to 3, there are 500 participants recruited. The decision to proceed between analysis 3 and analysis 4 considers the value in waiting for the remaining participants to complete follow-up (i.e., there are no trial costs but there is potential value lost by delaying the implementation of the best decision option). At analysis 3, $\mathrm{ENB}\;S^{j,j+1}$ is equivalent to $\mathrm{ENB}\;S^{j}$ . Recruitment and follow-up are completed by analysis 4.

**Figure 5:**
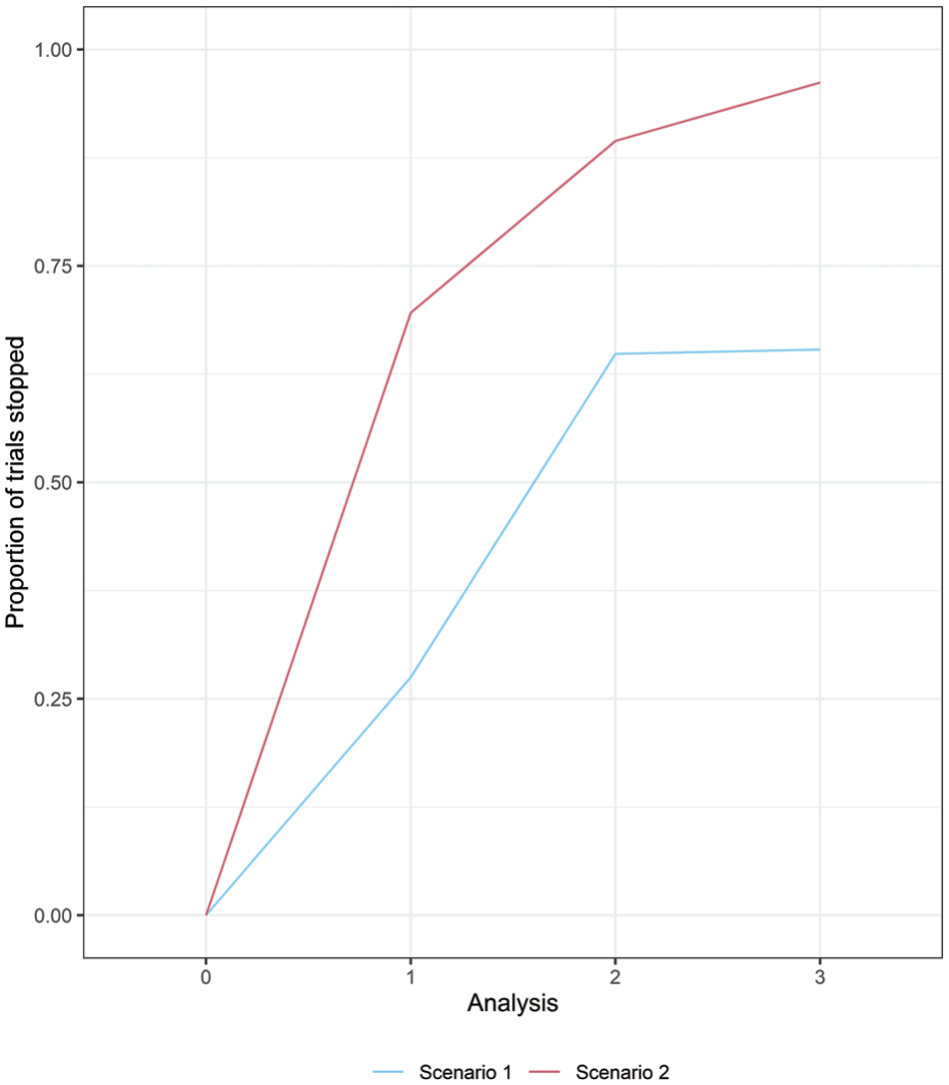
Proportion of 10,000 simulated trials stopping at each analysis in each scenario due to a negative expected net benefit of sampling ( $\mathrm{ENB}\;S^{j}$ ) in the respiratory syncytial virus case study.

**Figure 6:**
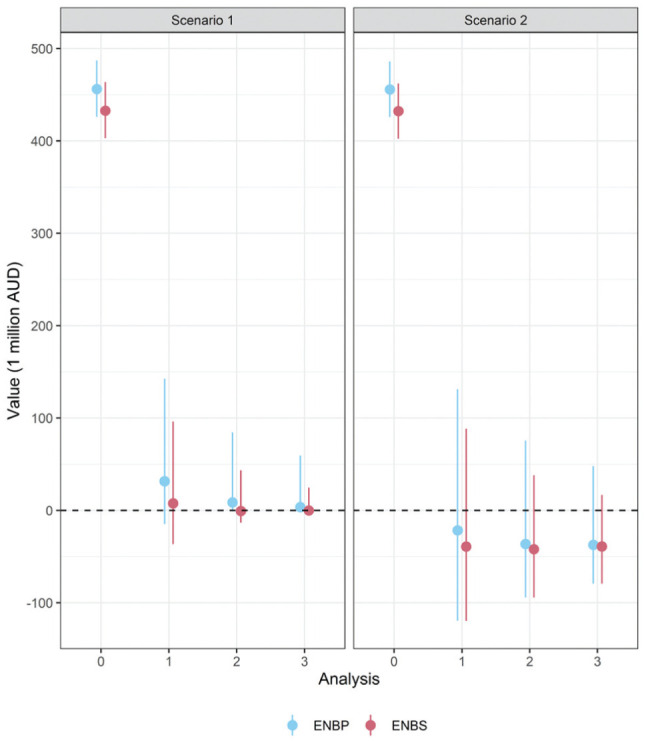
Median and 95% central interval of the expected net benefit of perfect information ( $\mathrm{ENB}\;P^{j}$ ) and sampling information ( $\mathrm{ENB}\;S^{j}$ ) across each analysis and each scenario for 10,000 simulated trials in the respiratory syncytial virus case study.

We conducted a simulation study investigating 10,000 simulated trials in each scenario. The proportion of simulated trials stopping at each analysis in each scenario is shown in Figure 5, and the distribution of $\mathrm{ENB}\;P^{j}$ and $\mathrm{ENB}\;S^{j}$ for the simulated trials at each analysis in each scenario are shown in Figure 6. The medians (interquartile range) for the $\mathrm{ENB}\;S^{j}$ run time in seconds were 0.73 (0.62, 0.91) and 1.05 (0.90, 1.19) for scenario 1 and scenario 2, respectively. The mean sample size recruited in scenario 1 and scenario 2 was 1,538 and 1,205 participants, respectively. The proportion of simulated trials that identified the optimal decision option at trial conclusion was 93.5% and 97.0% for scenario 1 and scenario 2, respectively.

## Discussion

VoI methods enable researchers to determine the net monetary value of their proposed research and therefore allocate resources more efficiently. Moreover, public research funders can use VoI methods to determine, in advance, which studies are most likely to be cost-effective and thereby set future research priorities and allocate limited research budgets accordingly.^56^ Recently, these methods have become accessible to researchers via a range of publicly available online tools,^57^ with their popularity coinciding with an increase in the advocacy of health economic considerations in general.

The value-driven adaptive design is fundamentally different from a traditional design (adaptive or not) in its philosophy, and therefore, we have chosen not to compare the designs. At the core of a traditional design is a hypothesis test that aims to determine whether or not a parameter (or set of parameters) exists within a region of interest (e.g., whether or not a treatment effect is zero). A resulting claim about the parameters of interest incurs the risk of being wrong, and consequently, type I error and power become important. Unfortunately, many researchers believe that the value of a trial lies in its ability to navigate this hypothesis test (e.g., trials that do not declare a nonzero treatment effect are often deemed to have “failed” or else are “inconclusive”). Trials with traditional designs may continue long after we are confident of the economic decision or alternatively may stop when the economic decision is still unclear.

In contrast, the value-driven adaptive design is centered around the (monetary or health) value of data gathered to inform a decision with respect to a decision model. It is not subject to type I error and power simply because it does not make a binary truth claim and instead focuses on reducing parameter uncertainty to improve the probability the decision maker makes an optimal decision. Of course, a suboptimal decision may still be made (akin to a type I or type II error), but by design, the trial stops at the point at which the incremental value to reduce the probability of making a suboptimal decision is outweighed by the cost of continuing the trial. Therefore, whether or not the optimal decision was chosen is irrelevant; the cost of collecting more data to inform the decision exceeds the expected benefit from making a better informed decision, implicitly because those resources could be diverted to where the benefits returned are likely to be greater. Furthermore, not only will a clinical decision likely consider multiple outcomes that a hypothesis test avoids based on multiplicity concerns, but in most circumstances, a decision will need to be made whether or not a trial is conducted, and the “correctness” of the decision made may be impossible to determine, even in hindsight.

Decision-theoretic models have been developed in the past decade that optimally select stopping times given their model-specific assumptions.^44,46–48^ These models employ dynamic programming methods to optimize decision making while carefully managing potential pathological behavior in the VoI functions (e.g., paradoxical nonconcavity^58^). However, approaches using data-driven trial-based cost-effectiveness analyses may be inappropriate, and using the trial to inform a subset of the parameters of the full decision model may be a preferable alternative.^52^ In scenarios in which the trial does not contain all of the decision options in the economic model, the VoI calculation is still valid but will depend on only the relevant subset of parameters (e.g., if the parameters associated with the missing decision options are independent from the relevant subset of parameters, then their uncertainty will remain unchanged).

Our model-based method seeks to provide a pragmatic alternative to these data-driven methods and does not, by any definition, claim to select optimal stopping times. Clinical trials serve multiple stakeholders and are subject to ethical and practical constraints^33^ that, consequently, may preclude the implementation of an “optimal” design. For example, a pragmatically designed trial appropriately informed by the relevant stakeholders, including consumers, may equally allocate participants to all available interventions even though it may counterintuitively be optimal to collect information on only a subset of the available interventions.^58^ Further practical considerations that may preclude the implementation of an optimal model include the complexity of the statistical model, randomization, intercurrent events, and constraints on statistical and laboratory resources. Our value-driven adaptive design is not contingent on the absence of these considerations and is flexible in its implementation by design. The resulting trial will likely be of value to decision makers, even if its value is “suboptimal.” In fact, the capability to determine an optimal solution (e.g., via the methods of Pertile et al.^44^) is explicitly traded off with the relaxation of unrealistic assumptions that may hinder the use of VoI methods in adaptive designs.

A limitation of our methodology is that it is constrained to designs in which the decision faced at interim analyses is only one related to increasing the sample size, where other design elements are fixed (e.g., length of follow-up time). Trial designs that allow for the follow-up time of currently enrolled trial participants to be increased may benefit from alternative methods previously developed in this space.^34,35^ However, if the decision is made to increase the sample size, including at the start of the trial, the randomization ratio still needs to be chosen. If feasible, our method can be used to select an advantageous randomization ratio that targets recruitment to a decision option associated with higher parameter uncertainty in order to maximize the VoI collected from the next interim sample. Another potential limitation is that it may be infeasible to compute $\mathrm{ENB}\;S^{j,j^{\;'}}$ when the statistical model or net benefit function are complex. In this situation, if $\mathrm{ENB}\;S^{j}<\epsilon <\mathrm{ENB}\;P^{j}$ , then the decision to proceed to the next analysis is complicated, and the optimal solution is not determinable.

Adaptive designs, including the value-driven adaptive design, are not suitable for all trials. For example, if the length of follow-up to outcome ascertainment is sufficiently long compared with the speed of enrolment, then the potential benefits of an adaptive design are limited (i.e., if there are few participants yet to recruit at the time of an analysis, then the ongoing trial design has limited opportunity to “adapt”). In these situations, a fixed design that is still based on VoI is recommended.

The RSV case study demonstrated the implementation of a simple value-driven adaptive design. A real-world implementation of this design would need to address the costs of implementation for each strategy as well as their expected uptake. Furthermore, the policy maker will likely be interested in additional outcomes (e.g., hospitalization, mortality, and adverse events) and will be required to specify an appropriate willingness-to-pay parameter that considers the full decision model, potentially specified in terms of QALYs. Moreover, for the case study, we assumed a simple research cost function, η(j) , based on a fixed per-dyad recruitment cost. In realistic scenarios, this function should be flexibly defined to incorporate varying recruitment costs (e.g., site-specific recruitment costs) and additional costs (e.g., statistical resources).

An important avenue for future work will be to explore the implementation of the value-driven adaptive design for a clinical trial including the potentially challenging formulation and agreement of a decision model and specification the research cost function. Arguably, this area of research, or at least its implementation, is still in its infancy, and further investigation and development is needed.

## Supplemental Material

sj-pdf-1-mdm-10.1177_0272989X261423177 – Supplemental material for A Pragmatic Bayesian Adaptive Trial Design Based on the Value of Information: The Value-Driven Adaptive Design Supplemental material, sj-pdf-1-mdm-10.1177_0272989X261423177 for A Pragmatic Bayesian Adaptive Trial Design Based on the Value of Information: The Value-Driven Adaptive Design by Michael Dymock, Julie A. Marsh, Mark Jones, Anna Heath, Kevin Murray and Thomas L. Snelling in Medical Decision Making
