# Supplementary material for: A Pragmatic Bayesian Adaptive Trial Design Based on the Value of Information: The Value-Driven Adaptive Design
Source: Med Decis Making. 2026 Mar 13;46(5):560–74. doi: 10.1177/0272989X261423177 (PMC13242537; doi:10.1177/0272989X261423177)
Supplement: sj-pdf-1-mdm-10.1177_0272989X261423177 – Supplemental material for A Pragmatic Bayesian Adaptive Trial Design Based on the Value of Information: The Value-Driven Adaptive Design [file sj-pdf-1-mdm-10.1177_0272989X261423177.pdf]

A pragmatic Bayesian adaptive trial design based on the value of information: the value-driven adaptive design

Supplementary Materials

In order to derive $\text{ENBS}^{j,j+1}$ (equation 5) from the main text we first introduce shorthand notation for the net benefit function evaluated for decision option d between times t_j and $t_{j'}$, the value accrued in the general population between times t_j and $t_{j'}$ (the current implementation of the interventions), and the research cost function for analysis j:

$$\text{NB}^{d}_{j,j'} = \text{NB}(d, \boldsymbol{\Theta}, t_j, t_{j'}) \tag{S1}$$

$$\mathbf{NB}^{\boldsymbol{\rho}}_{j,j'} = \sum_{d=1}^{D} p_d \times \text{NB}(d, \boldsymbol{\Theta}, t_j, t_{j'}) \tag{S2}$$

$$\eta_j = \eta(j) \tag{S3}$$

All expectations defined hereafter are conditional on $\boldsymbol{x}^j$ so we suppress the notation for simplicity (i.e., $\text{E}_{Z|\boldsymbol{x}^j}[\cdot] \equiv \text{E}_Z[\cdot]$ where Z is a random variable). We then define the random variable $\text{ENBS}^{j+1}|\boldsymbol{X}^{j+1}, \boldsymbol{x}^j$ as:

$$\begin{aligned} \text{ENBS}^{j+1}|\boldsymbol{X}^{j+1}, \boldsymbol{x}^j \equiv \text{ENBS}^{j+1}|\boldsymbol{X}^{j+1} = \; & \text{E}_{\boldsymbol{\Theta}|\boldsymbol{X}^{j+1}}\left[\mathbf{NB}^{\boldsymbol{\rho}}_{j+1,j+2}\right] + \\ & \text{E}_{\boldsymbol{X}^{j+2}|\boldsymbol{X}^{j+1}}\left[\max_d \text{E}_{\boldsymbol{\Theta}|\boldsymbol{X}^{j+1},\boldsymbol{X}^{j+2}}[\text{NB}^{d}_{j+2,H}]\right] - \\ & \left(\max_d \text{E}_{\boldsymbol{\Theta}|\boldsymbol{X}^{j+1}}\left[\text{NB}^{d}_{j+1,H}\right] + \eta_{j+2}\right) \end{aligned} \tag{S4}$$

Finally, using (S1)-(S4), we derive $\text{ENBS}^{j,j+1}$, the expected net benefit of sampling to analysis $j+1$ with the possibility of sampling to $j+2$ (if valuable), as follows:

$$
\begin{aligned}
&\mathrm{ENBS}^{j,j+1} = \\
&\mathrm{E}_{\boldsymbol{\Theta}}\left[\mathbf{NB}^{\boldsymbol{\rho}}_{j,j+1}\right] - \left(\max_d \mathrm{E}_{\boldsymbol{\Theta}}\left[\mathrm{NB}^d_{j,H}\right] + \eta_{j+1}\right) + \\
&\mathrm{E}_{\boldsymbol{X}^{j+1}}\left[\max\left\{\left(\max_d \mathrm{E}_{\boldsymbol{\Theta}|\boldsymbol{X}^{j+1}}[\mathrm{NB}^d_{j+1,H}]\right), \left(\mathrm{E}_{\boldsymbol{\Theta}|\boldsymbol{X}^{j+1}}\left[\mathbf{NB}^{\boldsymbol{\rho}}_{j+1,j+2}\right] + \mathrm{E}_{\boldsymbol{X}^{j+2}|\boldsymbol{X}^{j+1}}\left[\max_d \mathrm{E}_{\boldsymbol{\Theta}|\boldsymbol{X}^{j+1},\boldsymbol{X}^{j+2}}[\mathrm{NB}^d_{j+2,H}]\right] - \eta_{j+2}\right)\right\}\right] = \\
&\mathrm{E}_{\boldsymbol{\Theta}}\left[\mathbf{NB}^{\boldsymbol{\rho}}_{j,j+1}\right] - \left(\max_d \mathrm{E}_{\boldsymbol{\Theta}}\left[\mathrm{NB}^d_{j,H}\right] + \eta_{j+1}\right) + \\
&\mathrm{E}_{\boldsymbol{X}^{j+1}}\left[\max_d \mathrm{E}_{\boldsymbol{\Theta}|,\boldsymbol{X}^{j+1}}[\mathrm{NB}^d_{j+1,H}] + \max\left\{0, \left(\mathrm{E}_{\boldsymbol{\Theta}|\boldsymbol{X}^{j+1}}\left[\mathbf{NB}^{\boldsymbol{\rho}}_{j+1,j+2}\right] + \mathrm{E}_{\boldsymbol{X}^{j+2}|\boldsymbol{X}^{j+1}}\left[\max_d \mathrm{E}_{\boldsymbol{\Theta}|\boldsymbol{X}^{j+1},\boldsymbol{X}^{j+2}}[\mathrm{NB}^d_{j+2,H}]\right] - \left(\max_d \mathrm{E}_{\boldsymbol{\Theta}|\boldsymbol{X}^{j+1}}[\mathrm{NB}^d_{j+1,H}] + \eta_{j+2}\right)\right)\right\}\right] = \\
&\mathrm{E}_{\boldsymbol{\Theta}}\left[\mathbf{NB}^{\boldsymbol{\rho}}_{j,j+1}\right] + \mathrm{E}_{\boldsymbol{X}^{j+1}j}\left[\max_d \mathrm{E}_{\boldsymbol{\Theta}|\boldsymbol{X}^{j+1}}[\mathrm{NB}^d_{j+1,H}] + \max\left\{0, \mathrm{ENBS}^{j+1}|\boldsymbol{X}^{j+1}\right\}\right] - \left(\max_d \mathrm{E}_{\boldsymbol{\Theta}}\left[\mathrm{NB}^d_{j,H}\right] + \eta_{j+1}\right) = \\
&\mathrm{E}_{\boldsymbol{\Theta}}\left[\mathbf{NB}^{\boldsymbol{\rho}}_{j,j+1}\right] + \mathrm{E}_{\boldsymbol{X}^{j+1}}\left[\max_d \mathrm{E}_{\boldsymbol{\Theta}|\boldsymbol{X}^{j+1}}[\mathrm{NB}^d_{j+1,H}]\right] + \mathrm{E}_{\boldsymbol{X}^{j+1}}\left[\max\left\{0, \mathrm{ENBS}^{j+1}|\boldsymbol{X}^{j+1}\right\}\right] - \left(\max_d \mathrm{E}_{\boldsymbol{\Theta}}\left[\mathrm{NB}^d_{j,H}\right] + \eta_{j+1}\right) = \\
&\mathrm{ENBS}^{j} + \mathrm{E}_{\boldsymbol{X}^{j+1}}\left[\max\left\{0, \mathrm{ENBS}^{j+1}|\boldsymbol{X}^{j+1}\right\}\right]
\end{aligned}
\tag{S5}
$$
